# Propolis: a new frontier for wound healing?

**DOI:** 10.1186/s41038-015-0010-z

**Published:** 2015-07-22

**Authors:** Simona Martinotti, Elia Ranzato

**Affiliations:** DiSIT—Dipartimento di Scienze e Innovazione Tecnologica, University of Piemonte Orientale, “Amedeo Avogadro”, viale Teresa Michel, 11-15121 Alessandria, Italy

**Keywords:** Propolis, Wound healing, Tissue regeneration

## Abstract

Propolis is a resin produced by honeybees by mixing wax, pollen, salivary secretions, and collected natural resins.

The precise composition of propolis varies with the source, and over 300 chemical components belonging to the flavonoids, terpenes, and phenolic acids have been identified in propolis. Moreover, its chemical composition is subjected to the geographical location, botanical origin, and bee species.

Propolis and its compounds have been the focus of many works due to their antimicrobial and anti-inflammatory activity; however, it is now recognized that propolis also possesses regenerative properties.

There is an increasing interest in the healing potential of natural products, considering the availability and low cost of these products. Propolis contains a huge number of compounds that explicate some biological effects that speeds up the healing process and is widely used in folk remedies.

This review aims to condense the results on the mechanism of activity of propolis and its compounds.

## Introduction

Propolis is a generic name of a mixture of resinous substances collected by honeybees from parts of plants, buds, and exudates in the north temperate zone, extending from the Tropic of Cancer to the Arctic Circle.

The main sources of propolis are poplar, willow, birch, elm, alder, beech, conifer, and horse-chestnut trees [1].

Propolis is normally utilized in the hive to coat the inner walls, to shelter from the entrance of intruders, such as snakes and lizards, or against wind and rain, and to prevent fungi and bacteria growth [2].

A bee’s colony is able to collect from 150 to 200 g of propolis in 1 year; however, some colonies collect less than that [1].

Aristotle has coined the word propolis from the Greek words pro (before) and polis (city), meaning, before the city or defender of the city. Aristotle showed a remarkably accurate and detailed knowledge of propolis.

New evidence suggests that propolis in honey bee colonies may play a more subtle role in colony level immunity, than direct defense against parasites and pathogens [3].

### History of propolis

The long history of bee domestication has led to an exploitation of bee products, and the many favorable properties of propolis lend to its application in many human pursuits. Propolis is as old as honey, and man has used it for ages. Today, the long history of the use of propolis continues in home remedies and personal products [2].

There are records suggesting the use of propolis by ancient Egyptians, Persians, and Romans to alleviate many ailments and as an “embalming” substance.

The great consideration of bees and bee’s product from Egyptians is attested by the fact that one of pharaohs’ titles was “Bee King.” The ancient Jews considered tzori (the Hebrew word for propolis) as a medicine. Propolis has been utilized by Greeks as the primary ingredient of polyanthus, perfume which combined propolis, olibanum, styrax, and aromatic herbs.

Hippocrates, a Greek physician, considered the father of modern medicine, recognized the healing properties of propolis [4]. In addition, the Roman Pliny the Elder in his famous Natural History wrote about the properties of propolis showing a detailed understanding of propolis in the beehive. Pliny wrote also extensively about preventing wine from turning to vinegar by adding tree resins. Pine, cedar, and often terebinth were added to Roman wines for this purpose. Recent studies have shown that certain tree resins, like propolis, can kill bacteria, protecting from degradation organic compounds.

In the Middle Ages, propolis was not a very popular topic and its use as ointment disappeared. The interest for propolis turned back with the Renaissance interest for the recovery of old and forgotten treatments and approaches.

Antiquarian non-personal product or medicinal applications include propolis use in Italy in the 17th century, when Stradivari used propolis as an ingredient in the varnish of his instruments [5]. Today, propolis is still used with musical instruments in rosin for stringed instruments and in the repair of accordions [6].

## Review

### Characteristics of propolis

Nectar and pollen, the main materials collected by honeybees, are referred to by their botanical names. “Propolis” is a bee-oriented term that does not have a botanical derivation. Bees can use different materials for “manufacturing” of propolis, and these materials are produced in different botanical parts of plants.

These are substances actively secreted by plants and substances exuded from wounds in plants: lipophylic materials on leaves and leaf buds, mucilages, gums, resins, latices, etc. [7].

At elevated temperatures, propolis is soft, pliable, and very sticky; however, when cooled and particularly when frozen or at near freezing, it becomes hard and brittle.

Propolis becomes liquid at 60 to 70 °C, but for some samples, the melting point may be as high as 100 °C. Color of propolis varies from green to brown and reddish, depending on its botanical source.

The propolis’ composition as well as its color and aroma change according to the geographical zones.

Generally, ethanol is the best solvent for propolis preparation, and other solvents such as ethyl ether, water, methanol, and chloroform may be used for extraction and identification of propolis compounds [8].

Other solvents such as glycerin, propylene glycol, and other solutions have been used for propolis preparation for pharmaceutical and cosmetic industries [9].

Propolis obtained from hives, or rude propolis, contains around 50 % balsam resin, 30 % wax, 10 % essential and aromatic oils, 5 % pollen, and 5 % other substances, including wood fragments [5].

More than 300 different compounds have been characterized so far in propolis, including aliphatic acids, esters, aromatic acids, fatty acids, carbohydrates, aldehydes, amino acids, ketones, chalcones, dihydrochalcones, terpenoids, vitamins, and inorganic substances. Of all, flavonoids are the compounds that possess greater research interest [10].

The positive activities of propolis are more numerous in tropical regions than in temperate climates, reproducing the richer vegetal diversity observed in the former [11].

However, such properties can change depending on the composition and polyphenol content that in turn depend on several factors including season, vegetation of the area, geographical origin, and the state of propolis (fresh or aged) [12].

The composition of propolis is guided by the phyto-geographic characteristics of beehive surroundings [13], see also Table 1. Seasonal variations occurring within the same place has been described. Some authors have also reported variations among samples collected in the same area but by different *Apis mellifera* subspecies [14].

**Table 1:** Most important propolis types: geographical origin and major constituents

Propolis type	Origin	Major components	Ref.
Poplar	Europe, North America	Polyphenols	[15–18]
Red propolis	Cuba, Mexico, Brazil	Prenylated benzophenones	[19–21]
Mediterranean propolis	Greece, Malta, Crete, Southern Italy	Diterpenes	[23, 24]
Pacific	Taiwan, Japan	C-Prenyl-flavanones	[20]

Propolis collected from many countries (such as China, Korea, Croatia, New Zealand, and Africa) [15–18] showed chemical composition similar to poplar. In fact, poplar tree is a typical tree in Europe and is used to name the common type of propolis. Therefore, the current opinion is that bees collect propolis from resins of poplars and conifers. This propolis is characterized by high level of flavonones, flavones, low phenolic acids, and their esters.

However, in some areas, poplars are not native plants, such as in Australia and South America and bees seek out other plants to produce propolis, which has a similar composition to the poplar propolis.

Propolis from the tropical area, such as Brazilian green and red propolis, are rich respectively in prenylated derivatives of p-coumaric acid and some isoflavonoids that are different from the ones found in poplar propolis [19].

For the propolis of the southeast Brazil, *Baccharis dracunculifolia* is the main botanical source and the Artepillin C, as the most peculiar component, makes it easy to discriminate from other type of propolis [20, 21].

It has also been reported that propolis from Venezuela, Amazon, and Cuba contains prenylated benzophenones, which are originated from exudates of *Clusia* flower [22].

*Macaranga* plants have been established to be the font of propolis in Taiwan and Japan [20]. High concentrations of diterpenoids in Mediterranean basin propolis may originate from *Cupressus* and *Pinus* plants [23, 24].

However, some of plant sources are just hypothesized by observing the foraging behaviors of bees and not comparing the chemical composition of secondary metabolites in propolis and in the plant source. So, there is a strong need to compare the chemical composition of propolis and of plants to confirm the exact botanical origin [25].

Some contaminants such as pesticide, copper, iron, magnesium, and even lead can be collected by bees and added to propolis [26].

### Biological effects of propolis compounds

An inclusive spectrum of positive biological activity of propolis with respect to the human body largely results from the anti-oxidative effects of polyphenols [27]. Unsettling of the balance between production and deactivation of reactive oxygen species (ROS) leads to many disorders. Free radicals can oxidize cell proteins, nucleic acids, and lipids.

The mechanisms of the anti-oxidative activity of polyphenols are different, such as the ability of inhibiting the appearance of ROS, chelating ions of metals involved in the ROS creation, and scavenging ROS, thus interfering with the cascade of reactions leading to the peroxidation of lipids and synergistic cooperation with other antioxidants.

Intriguingly, propolis displays also anti-inflammatory properties in both acute and chronic inflammatory processes, and this is principally due to its large content of polyphenol compounds.

Propolis contains also active compounds which are known to promote cell proliferation or apoptosis. Among them, there are caffeic acid, caffeic phenyl ester, artepillin C, quercetin, resveratrol, galangin, and genistein [28, 29].

Genistein, quercetin, kaempferol, luteolin, chrysin, and apigenin inhibit cyclins, arresting the cell cycle [30]. Galangin, genistein, and resveratrol display antiproliferative activity with respect to the breast cancer estrogen receptor [31].

Some in vivo tests have demonstrated that some propolis flavonoids inhibit the development of lung cancer and oral cancer, as well as skin, esophagus, stomach, colorectal, prostate, and breast cancers [32].

Flavonoids are considered as valuable natural compounds not only because they avoid rapid blood sugar rises in the serum, but also because they are able to shelter diabetics from the complications of this metabolic disorder.

Matsui et al. [20] observed for Brazilian propolis anti-hyperglycemic effects with respect to caffeoylquinic acids (CQA). Caffeoylquinic acids are powerful inhibitors of β-glucosidase and α-amylase.

Another author [33] demonstrated that, in rat with diabetes, the administration of propolis extracts leads to a reduced level of glucose and a protective effect against lipid peroxidation.

**Fig. 1:**
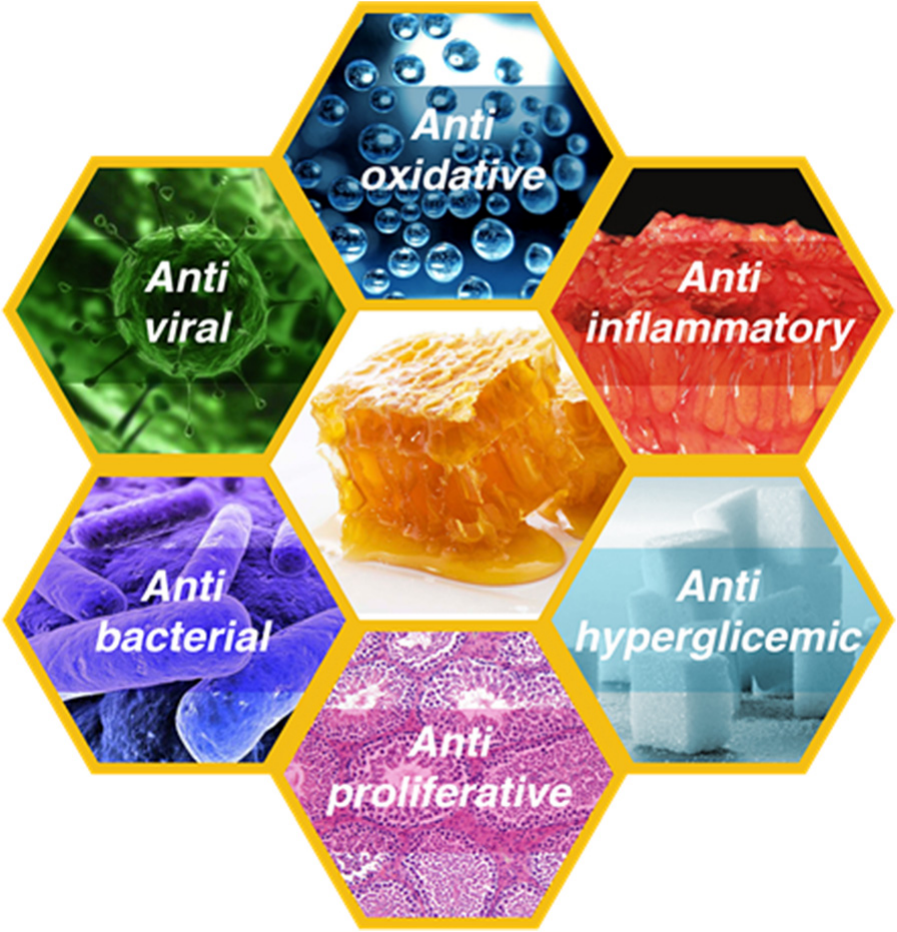
Molecular mechanisms/targets mediating effects of propolis

Many works have demonstrated the important antibacterial actions of propolis, where much greater activity was determined with respect to Gram-positive bacteria than to Gram-negative ones [34]. These antibacterial effects may due to the synergistic activity of the many compounds present in propolis. Pinocembrin displays an intense antibacterial activity against *Streptococcus* sp. Apigenin most powerfully inhibits bacterial glycosyltransferase. p-Coumaric acid, artepillin C, and 3-phenyl-4-dihydrocinnamylocinnamic acid are effective against *Helicobacter pylori* [35, 36] Fig. 1.

### Use of propolis in experiments

Some techniques such as high-performance liquid chromatography (HPLC), gas chromatography (GC), as well as identification techniques, such as mass spectroscopy (MS), gas chromatography, and mass spectroscopy (GC-MS) allowed to increase the number of compounds present in propolis [25].

Therefore, many papers regarding the different roles and different biological properties of propolis’ components have been recently published.

However, a huge part of them are of limited usefulness, for the lack of source for comparison and scientific assessment of the results and because they do not refer to the chemical composition of the studied propolis.

The utilization for research purpose of characterized propolis samples and for comparative study of positive effects of propolis from different geographic areas is the most interesting methodologies in the recent research.

Recently, chemical characterization of the samples tested for antibacterial activity was compared with quantification of the major groups of biologically active substances of the corresponding propolis samples [36]. The results confirm the importance of phenolic acids for propolis antibacterial activity, and the significance of poplar as a propolis source, which offers the most efficient defense for hives against bacteria.

Banksota et al. compared the cytotoxic, hepatoprotective, and free radical scavenging activity of propolis from Brazil, Peru, The Netherlands, and China. The authors found that propolis from The Netherlands and China possessed the strongest cytotoxic activity; while almost all samples possessed significant hepatoprotective activity [37].

The work of Kumazawa et al. [38] is another example of this approach. The authors linked the antioxidant activity of propolis (Argentina, Austria, Brazil, Bulgaria, Chile, China, Hungary, New Zealand, South Africa, Thailand, Ukraine, Uruguay, the USA, and Uzbekistan) and combined these data with chemical analyses.

Major components of propolis were recognized by HPLC analysis with photo-diode array and mass spectrometric detection and quantitatively analyzed. Propolis extracts from Argentina, Australia, China, Hungary, and New Zealand had relatively strong antioxidant activities and were associated with the total polyphenol and flavonoid contents. In fact, the propolis with strongest antioxidant activity contained anti-oxidative compounds such as kaempferol and phenethyl caffeate.

Following a similar approach, Chen et al. related the radical scavenging activity, cytotoxic effects and apoptosis induction in some human melanoma cell lines of propolis from different localities of Taiwan [17].

### Wound healing properties of propolis

Along with other honeybee products (honey, royal jelly, pollen), propolis has great therapeutic properties, being used since antiquity in popular medicine in various parts of the world [39, 40].

Propolis is believed to have antiseptic, antibacterial, antimycotic, astringent, spasmolytic, anti-inflammatory, anesthetic, antioxidant, antifungal, antiulcer, anticancer, and immunomodulatory effects [41, 42], see Table 2.

**Table 2:** Most important properties of propolis

Effects	Ref.
Antibacterial, anti-viral, and anti-fungin	[9, 34–36]
Antioxidant	[45, 46]
Anti-inflammatory	[41, 42, 46]
Anti-tumoral	[42, 59–61]
Anti-cariogenic	[57, 58]
Wound healing	[43, 48–50]

Propolis, which is well tolerated with rare incidents of allergy and no toxicity, is referred to as an excellent candidate for burn management, enhancing skin cell proliferation, activation, and growth capacity [43].

Some results confirm the propolis therapeutic efficacy, throughout quantitative and qualitative analyses of collagen types I and III expression and degradation in wounds matrix, indicating that propolis could have favorable biochemical environment supporting re-epithelization [44].

Recent discoveries have identified that oxygen is required to disinfect wounds and fuel healing but also that oxygen-dependent redox-sensitive signaling processes plays a pivotal role in the repair cascade.

The interactions of free radicals in skin and in neighboring tissues may be also responsible for some toxic effects and modifications of their structures.

A recent report [45] suggested that propolis is able to quench free radicals in skin. This outcome of propolis on free radicals in the epidermis is the source of safety of its application in the therapy of burn wounds.

Other findings reveal that propolis speed up the burned tissue repair by stimulation of the wound bed matrix remodeling, proposing that the observed changes in extracellular matrix content after propolis application may be connected with the ability of its flavonoid compounds to reduce lipid peroxidation and to prevent necrosis of cells [44].

Biological activities of propolis on wound repair and tissue regeneration might be correlated to its antimicrobial, anti-inflammatory, and immumonodulatory properties [46].

Propolis has demonstrated some in vitro antimicrobial activity, in particular against Gram-positive (*Staphylococci* and *Streptococci* spp.) and Gram-negative bacteria (*Escherichia coli*, *Klebsiella pneumoniae*, *Proteus vulgaris*, and *Pseudomonas aeruginosa*), *Helicobacter pylori*, protozoa (*Trypanosoma cruzi*), fungi (*Candida albicans*), and viruses (such as HIV, Herpes viruses, or influenza viruses).

Antimicrobial properties of propolis are essentially due to the flavonoid content and in particular to the presence of pinocembrin, galangin, and pinobanksin. Pinocembrin also exhibits antifungal properties. Other compounds with well-established effects are ester of coumaric and caffeic acids.

Some studies have highlighted the role of propolis as the solvent employed for the extraction of propolis that may influence the potency of its antimicrobial activity [9].

Propolis also shows anti-inflammatory effects against acute and chronic models of inflammation, but how propolis induces this effect is still unclear. Rossi et al. [47] verified that propolis inhibits, in a concentration-dependent manner, COX activity from lung homogenates of saline- or LPS-treated rats [47].

Propolis demonstrates immunostimulatory and immunomodulatory effects in vitro on macrophages; while in vivo it increases the ratio of CD4/CD8 T cells in mice [48].

The results of this study [48] show that application of propolis increases the wound healing rate and re-epithelialization of diabetic wounds in rodents. It has also proposed other roles for propolis in decreasing neutrophil infiltration and normalizing macrophage influx into wounded area.

Wound repair and regeneration proceeds via a finely tuned pattern of integrated phases, such as hemostasis, inflammation, cell proliferation, and tissue remodeling, which all involve a number of cellular and molecular processes [49]. This phenomenon includes migration and proliferation of epidermal cells and keratinocytes, fibroblast adherence, and extracellular matrix (ECM) contraction [50]. Propolis treatment stimulates significant increases in ECM components during the initial phase of wound repair, followed by a reduction in the ECM molecules. It is postulated that this biological effect of propolis is associated with its ability to stimulate the expression of transforming growth factor-β (TGF-β) [51] that participates in the early phases of wound repair such as hemostasis and inflammation.

McLennan and coworkers [52] showed that with a single propolis topical, there is an increase of wound healing in a diabetic rodent model of full-thickness cutaneous wound healing. This was the first systematic study showing that propolis improve wound healing in diabetes.

Some works have explored the effects of propolis solutions at animal wounds treatment in clinical and experimental cases [53]. The results showed that propolis is able to perform a good healing process mainly by reducing the inflammatory response; so, healing process was faster with propolis. The authors considered propolis suitable for wound treatment, following eradication of the infection.

The healing properties of propolis should be also due to its immune stimulating effect. This property was characterized in few clinical studies. Propolis was given, and cytokine secretion capacity was considered during and after treatment. The cytokine secretion capacity increased significantly during the treatment period in a time-dependent manner. The authors concluded that propolis was able to elicit an immune reactivity without side effects [54].

In the last decades, there was a bigger interest in the use of biomaterials, for example biopolymers, in healthcare products, especially as dressing for wounds, a fact that is predominantly associated to the renewable nature, biocompatibility, and biodegradability of these supplies. Tissue engineering needs polymeric membrane for creating the correct environment for cell migration and attachment within the scaffold. Therefore, biocompatible propolis loaded polyurethane nanofibers were successfully prepared using electrospinning of propolis solution [55].

### Other properties of propolis

Propolis has been proposed as anti-calculus agents, because it decreases the formation of oral calcium-phosphate precipitate (the main component of dental calculus).

Hidaka and colleagues [56] demonstrated that some types of honeys and propolis are able to reduce the amorphous calcium-phosphate transformation rate into hydroxyapatite.

The activity of propolis against oral bacteria has been explored, suggesting the effectiveness of propolis as an anti-cariogenic product [57].

Samples of saliva of 25 human healthy subjects and 25 patients affected by chronic periodontitis were analyzed for the extent of the microbial inhibition zone, comparing propolis and chlorhexidine efficacy. Propolis exhibited significant antibacterial activity against bacteria in both healthy and pathological saliva [58].

With an increasing incidence rate of cancer worldwide, new anticancer agents are still mandatory. The results of in vitro and in vivo studies suggest that propolis possess important cytotoxic properties against cancer cells through the induction of apoptosis or cell division and cell growth arrest [59–61].

Propolis and its compounds may inhibit cell cycle proliferation or induce apoptosis in cancer cells. They induce the apoptosis pathway by stimulation of Bax, p53, p21 proteins, p38 MAPK, JNK, ERK kinases, cytochrome c release, and activation of caspase cascade [42].

Although many studies have revealed the inhibitory effects of propolis and its compounds on growth and tumor propagation, further investigation is necessary to appreciate the efficiency and mechanism of their beneficial properties [42, 62].

## Conclusions

The valuable properties of propolis are revealed in the writings of ancient Greek and Roman physicians. Propolis is a non-toxic natural compound, and the safe concentration for human would be approximately 1.4 mg/kg day or 70 mg/day [63]. Some cases of allergy and contact dermatitis to propolis have been described [64], mainly among beekeepers [65].

Propolis contains a wide-ranging spectrum of chemical compounds that have many biological actions. It is believed a useful product and is already used in alternative medicine.

Other studies on the active components of propolis are strongly needed in order to identify interactions mediating their biological effects.

More works are also necessary on propolis’ component bioavailability, stability in different preparations, and safe and effective doses for management of disease.
